# Editorial: The Immunology of Sepsis—Understanding Host Susceptibility, Pathogenesis of Disease, and Avenues for Future Treatment

**DOI:** 10.3389/fimmu.2020.01263

**Published:** 2020-06-23

**Authors:** Luregn J. Schlapbach, Johannes Trück, Thierry Roger

**Affiliations:** ^1^Paediatric Critical Care Research Group, Paediatric Intensive Care Unit, Child Health Research Centre, Queensland Children's Hospital, The University of Queensland, Brisbane, QLD, Australia; ^2^University Children's Hospital Zurich and University of Zurich, Zurich, Switzerland; ^3^Lausanne University Hospital and University of Lausanne, Epalinges, Switzerland

**Keywords:** immune response, infection, organ dysfunction, personalized medicine, sepsis, septic shock, dysregulated host response

Sepsis is defined as a life-threatening organ dysfunction caused by a dysregulated host response to infection (1). Most recent estimations suggest that sepsis affects about 49 million people and accounts for around 11 million deaths each year worldwide (2), making it one of the leading causes of preventable death in all age groups. Sepsis is a health priority according to the World Health organization (3), which provided recommendations to improve the prevention, diagnosis and management of sepsis. Although sepsis mortality has decreased over the past two decades (4, 5), the incidence of sepsis continues to rise due to a higher number of patients with complex conditions such as immunosuppression as well as an increase in more elderly people. A basic requirement for effective sepsis management is timely and appropriate antimicrobial therapy (6, 7). With the increase of infections caused by multidrug resistant bacteria, the advances made toward rapid multidrug testing to enable personalized antimicrobial therapies is promising (Sun et al.). However, a major obstacle to improving sepsis outcomes is the lack of knowledge on the intricate interplay between host defense, infection, and pathogen virulence, as well as timing and nature of interventions. Over the past decade, our understanding of sepsis has evolved from the earlier concept of sepsis as a result of an excessive inflammatory response to the current notion that sepsis outcomes are more likely related to the dynamic balance between pro-inflammatory and counteracting anti-inflammatory mechanisms. To this end, animal models and a number of human observational studies have paved the way toward precision trials on immunotherapy for sepsis (Zijlstra et al.).

The aim of the Frontiers in Immunology Research Topic “The Immunology of Sepsis–Understanding Host Susceptibility, Pathogenesis of Disease, and Avenues for Future Treatment” was to collect state-of-the art articles and reviews on the role of the host immune system affecting susceptibility, presentation, and outcome of sepsis. We hereby provide an overview of this Frontiers in Immunology topic which includes 11 original articles, 13 review articles, 1 commentary, and 1 case report.

## Susceptibility to Sepsis

Sepsis susceptibility may result from maturational, genetic, or acquired alterations of the immune system. The incidence of sepsis is highest in newborns (8), and an increasing body of studies characterize distinct patterns of immune responses in this age group compared to children and adults. Schneider et al. for example, demonstrate that neonatal macrophages express substantially less tumor necrosis factor compared to adult cells, regulated through the transcription factor interferon regulatory factor 5 (IRF5).

Considering how few patients develop sepsis in comparison to the exposed population, there is biological plausibility and epidemiologic evidence for underlying genetic mechanisms affecting susceptibility to sepsis (9, 10). These may affect both very rare variants associated with extreme phenotypes (11) and common variants that may be of relevance at the populational level (12). Deficiency in mannose-binding lectin (MBL) emerged two decades ago as a promising candidate to investigate this hypothesis (13). Although polymorphisms affecting MBL serum levels are common and the role of complement in host defenses against bacterial infections is well known, previous reports on the relevance of MBL deficiency in susceptibility to infection remained conflicting (14). Using a well-phenotyped prospective intensive care unit cohort, Levy et al., genotyped 420 pediatric patients with influenza-associated organ dysfunction for variants in the *MBL2* gene expected to result in low serum MBL levels. No clear relationship was observed between genetic variants and overall outcomes in the cohort, neither in within-cohort, nor in trio or control analyses. Interestingly, and similar to a previous study, low-MBL producing variants were more common in a subset of fatal cases with methicillin-resistant *Staphylococcus aureus* (MRSA), but the relevance of this finding remains to be confirmed in larger studies.

## Host-Pathogen Interaction

Children are prone to severe infections caused by encapsulated bacteria such as *Streptococcus pneumoniae, Streptococcus agalactiae* (Group B streptococcus, GBS), *Neisseria meningitis* and *Haemophilus influenzae*. The polysaccharide capsule is a key bacterial virulence factor hindering opsonophagocytosis and complement-mediated bacteriolysis. However, it also represents an effective target for vaccine development. In his review, Sadarangani describes the mechanisms of vaccine-induced protection and illustrates successful examples of pneumococcal and meningococcal capsular conjugate vaccines, while challenges remain in the development of effective vaccines against non-type b *H. influenzae* and GBS that also carry polysaccharide capsules. The article by Kadhim et al., demonstrates a novel potential drug candidate to block staphylococcus enterotoxin-induced organ damage. Finally, Deng et al. investigate mechanisms of complement evasion in streptococcal strains.

Very timely considering the current coronavirus disease 2019 (COVID-19) pandemic, and giving credit to an often overlooked problem, Lin et al., provide a thorough overview of the epidemiology and pathogenesis of viral sepsis with a focus on herpes simplex, influenza, and dengue viruses, as well as entero- and parechoviruses. Recognition of a viral origin of sepsis could allow targeted treatment and reduce the unnecessary use of antibiotics.

## Understanding the Dysregulated Host Response to Infection

While the concept of a “dysregulated host response to infection leading to organ dysfunction” in sepsis is broadly accepted, our understanding of the underlying mechanisms remains very limited. The contributions to this Frontiers topic shed light on a number of pathways that are likely involved.

Previous studies have shown that a subgroup of septic children and adults reveal similar patterns to patients with hemophagocytic lymphohistocytosis (HLH), or macrophage activation syndrome (MAS) such as cytokine storm, hyperferritinemia, and multi-organ dysfunction. This entity has been termed macrophage activation-like syndrome (MALS). Karakike et al. provide an overview of the available evidence on this topic, while the results of the first randomized clinical trials (RCTs) on MALS are eagerly awaited. Zarjou et al. assessed the inhibitory role of myeloid ferritin heavy chain and ferritin light chain in a mouse model on nuclear factor kappa-light-chain-enhancer of activated B cells (NF-kB) activation, illustrating a potential immunomodulatory role of ferritin light chain.

More recently, insight into pro- and anti-inflammatory responses to the exposure of pathogen- and damage-associated molecular patterns (PAMPs and DAMPs) suggest a dynamic and heterogenous process (15). A fascinating research area in this regard relates to epigenetic processes affecting gene expression during sepsis. Cross et al. provide an overview on the literature on this topic. They summarize the rapidly growing number of mainly laboratory studies indicating that epigenetic mechanisms are thoroughly perturbed during sepsis, and are related to endothelial dysfunction and immunosuppression. These observations may open up new treatment options, such as drug interventions with histone deacetylase inhibitors for which pre-clinical animal studies suggest a potential benefit. It will be interesting to see whether candidate drugs progress to clinical studies on immune modulation in sepsis in the coming decade. Another possible drug approach that could support a more balanced immune response is the blocking of the Src family of tyrosine kinases with dasatinib, a drug for the treatment of chronic myeloid leukemia and acute lymphoblastic leukemia. Indeed, Gonçalves-de-Albuquerque et al. report promising results of using dasatinib in a mouse model of polymicrobial sepsis.

Several other proteins and cells represent both encouraging biomarkers and therapeutic targets for future strategies to combat sepsis. For example, Schrijver et al. report that myeloid-derived suppressor cells (MDSCs) are a heterogenous group of immature cells that expand in a number of conditions including sepsis. MDSCs suppress immune responses of different cell types in the early and late phases of sepsis, and pre-clinical studies show distinct patterns of expansion of MDSC subpopulations Observational studies have already reported an association between high proportions of blood MDSCs and poor outcome in sepsis patients. In a mouse model, Bomans et al. have elucidated mechanisms involved in the functional reprogramming of naïve bone marrow monocytes after sepsis, which lead to a “memory” of the innate immune system.

Other articles submitted to this Research Topic illustrate a number of recent discoveries of cells and pathways that contribute to the host response to sepsis. Yin et al., demonstrate that phosphatase regenerating liver 2 (PRL2) regulates the generation of reactive oxygen species in macrophages and thereby contributes to bactericidal activity. Sjaastad et al., assessed the response to polymicrobial sepsis using a mouse model and observed features consistent with chronic immunoparalysis, measured by reduced antigen-specific T cell-dependent B cell responses.

Although the intestinal system has not been commonly considered in organ dysfunction scores (1), it represents an extraordinary body surface area containing a high density of lymphatic tissues and immune cells. The regulation between the host immune system and intestinal pathogens as well as bacterial translocation play a key role in the dynamic processes during and after shock. Increasing our understanding of these events may point to ways for secondary interventions, as summarized in the review by Haussner et al., on the role of the intestinal mucosa during sepsis. Expanding from this work, the importance of intestinal microbiota in sepsis is explored in the excellent review by Haak et al. shedding light on the therapeutic potential of microbiome-modifying strategies for prevention and treatment of sepsis and sepsis-related late mortality.

## Biomarkers, Monitoring the Immune System, and the Future of Immunomodulation in Sepsis

The immune status of patients with sepsis is subject to rapid progression, and hyperinflammation and immunoparalysis may change dynamically. The failures to characterize the predominant immunological phenotype of sepsis patients coupled with considerable disease and patient heterogeneity represent major obstacles to develop effective immunomodulation-based trials. Peters van Ton et al. discuss the critical need for accurate monitoring of associated immune dysregulation to select patients more likely to benefit from targeted immunomodulatory interventions, and to facilitate enrolment in clinical studies toward personalized medicine.

The initial response to infection includes activation of innate immunity through soluble and cell-bound pattern recognition receptors. Porte et al. for example, review the available evidence on the role of the long pentraxin 3 (PTX3) in sepsis, a molecule with high homology with C-reactive protein. PTX3 binds to various microorganisms and has protective effects against sepsis in animal models. In human studies, high blood levels of PTX3 are associated with sepsis severity, suggesting a potential use as a biomarker. Furthermore, Tipoe et al. performed a systematic review and meta-analysis of plasminogen activator inhibitor-1 (PAI-1) in sepsis. PAI-1 level is increased in patients, and may be used as a predictor of disease severity and all-cause mortality in sepsis.

Low expression levels of human leukocyte antigen D related (HLA-DR) by CD14 leukocytes are increasingly used as a marker of sepsis-induced immunosuppression. Tamulyte et al. report on a prospective cohort assessing monocyte HLA-DR by a point-of-care test. They demonstrate the principle feasibility of the approach, which may help to identify patients with a higher risk of worse outcomes due to sepsis. Excitingly, the development of novel assays for immune profiling patients with severe infections has made huge progress and is anticipated to enable personalized medicine trials in the very near future.

The most widely studied immunomodulatory drugs in sepsis are corticosteroids. A large number of high quality RCTs in critically ill adults are available, as nicely reviewed by Heming et al. The immunomodulatory effects of statins have been recognized for some time. Braga Filho et al. used a mouse model of CLP to demonstrate the positive impact on simvastatin on survival and immune modulation.

## Conclusions

In summary, the knowledge on the pathophysiology of sepsis has expanded in recent years. Many original and review article in this Research Topic describe experimental and clinical studies pointing to new candidates for biomarkers and interventions, which may ultimately pave the way toward personalized care for sepsis. The availability of improved immunophenotyping approaches, coupled with a number of candidate drugs currently under investigation is promising. The marked heterogeneity of sepsis advocates for highly selective interventions, targeted at populations selected for patients who are more likely to respond to, and to benefit from therapeutic interventions. The success of this endeavor will depend on the capability to incorporate effective strategies for patient selection and targeted treatment allocation. As a promising sign, a number of consortia have recently been launched to advance personalized immunotherapy in sepsis. As nicely discussed by Talisa et al. trials using adaptive enrichment and response adaptive randomization may be best suited to tackle the challenges of developing effective treatments for clinical practice. Recently, large international randomized, embedded, multifactorial adaptive platform trials (originally developed in the field of cancer clinical research) have been launched in infectious diseases and hopefully will boost the efficiency and impact of sepsis research. These advances may motivate further personalized medicine trials leading to sustainable outcome improvements for this major killer disease.

## Author Contributions

All authors contributed to the article and approved the submitted version.

## Conflict of Interest

The authors declare that the research was conducted in the absence of any commercial or financial relationships that could be construed as a potential conflict of interest.
